# Effect of Enamel Pretreatment with Pastes Presenting Different Relative Dentin Abrasivity (RDA) Values on Orthodontic Bracket Bonding Efficacy of Microfilled Composite Resin: In Vitro Investigation and Randomized Clinical Trial

**DOI:** 10.3390/ma15020531

**Published:** 2022-01-11

**Authors:** Maria Francesca Sfondrini, Maurizio Pascadopoli, Simone Gallo, Federico Ricaldone, Davide Dirk Kramp, Margherita Valla, Paola Gandini, Andrea Scribante

**Affiliations:** 1 Unit of Orthodontics and Pediatric Dentistry, Section of Dentistry, Department of Clinical, Surgical, Diagnostic and Pediatric Sciences, University of Pavia, 27100 Pavia, Italy; francesca.sfondrini@unipv.it (M.F.S.); federico.ricaldone01@universitadipavia.it (F.R.); davidedirk.kramp01@universitadipavia.it (D.D.K.); paola.gandini@unipv.it (P.G.); 2 Unit of Dental Hygiene, Section of Dentistry, Department of Clinical, Surgical, Diagnostic and Pediatric Sciences, University of Pavia, 27100 Pavia, Italy; margherita.valla01@universitadipavia.it

**Keywords:** orthodontics, bonding, brackets, enamel, pretreatment, toothpaste, RDA, shear bond strength, ARI, failure rate

## Abstract

Bonding failure is a clinical issue frequently encountered in orthodontic practice. The aim of this study was to evaluate enamel pretreatment both in vitro and clinically using agents with different RDA values before brackets’ bonding, to assess if RDA can affect the Shear Bond Strength (SBS), Adhesive Remnant Index (ARI) and clinical failure rate of orthodontic brackets. For the in vitro study, 220 bovine teeth were pretreated with agents with different RDA values. Subsequently, brackets were bonded. For the clinical study, 20 patients underwent bonding of 20 brackets each with a split-mouth design. Low and high RDA toothpastes were used for enamel pretreatment. SBS, ARI and failures were recorded. Higher SBS values were found for teeth pretreated with lower RDA agents; conversely, lower SBS values were found for teeth pretreated with higher RDA agents (*p* < 0.05). For high ARI values, RDA increased too (*p* > 0.05). In the clinical study, a significantly lower failure rate was reported for teeth pretreated with low RDA toothpaste (2.5% in low RDA group, 7.0% in high RDA group; *p* < 0.05). No significant differences were assessed comparing the two dental arches and anterior and posterior sites. Enamel pretreatment with low RDA toothpastes could increase brackets’ survival rate. Further in vitro and clinical studies would be welcomed to confirm these findings.

## 1. Introduction

The aims of orthodontic therapy are the improvement of oral health conditions and, as a consequence, better facial aesthetics, which are the reasons of the increasing request of treatment by adults [1]. Nowadays, different treatments are available: fixed appliances, with labial or lingual brackets, and clear aligners [2,3]. Focusing on labial fixed appliances, the main discomforting situations consist of oral mucosal lesions, such as erosions, desquamations and ulcerations caused by brackets and archwires [4,5]. In addition, breakages of wires and brackets detachments can occur [5]; the problem is relevant as 61% of re-called patients from all the considered cohorts reported a breakage of a fixed orthodontic appliance [6].

During orthodontic therapy, a common but unpleasant occurrence is the possibility of brackets’ detachment, which can cause the lengthening of treatment time and give discomfort to the patient [7,8,9,10,11]. A reasonable clinical failure rate should be below 10%, but effectively, it has been assessed to be in the range of 0.6–28% [12]. The reasons behind bonding failure lie in the design of the bracket’s base, in the surfaces of bonding and in their treatment methods [13,14]. Excluding the bracket-related factors, it is widely accepted that the gold standard for brackets’ bonding is enamel conditioning with 37% orthophosphoric acid [13]. However, a great variety of pretreating agents have been studied, in particular with the purpose of evaluating their role in the remineralization process [15,16,17]. In fact, enamel pretreatment is a procedure consisting of enamel polishing with the aim of removing the bacterial plaque and organic debris; therefore, the cleaned surface obtained improves brackets’ adhesion [18]. Specific abrasives are used, which act on enamel. These agents can be toothpastes (for domiciliary use), pastes (i.e., pumice) and powders (these latter two for professional use). Pretreating agents are divided according to their abrasion capacity on enamel and dentin, respectively with the following indexes: REA (Relative Enamel Abrasivity) and RDA (Relative Dentin Abrasivity) [19].

The Relative Dentin Abrasivity (RDA) index is the gold standard to assess the abrasive potential of toothpastes. It is determined under laboratory conditions exploiting sound radioactive dentin. The tested toothpaste is used to brush radioactive dentin a certain number of times, therefore causing the release of a certain quantity of radioactive dentin. The same procedure is performed with an abrasive standard, this latter having an arbitrary value of abrasivity of 100. Therefore, comparing the quantities of the released dentin of the two toothpastes, the abrasivity of the tested toothpaste is expressed as a percentage of the standard value [19]. The RDA range of toothpaste formulations is included in the range 30–250, with no possibility of using products whose abrasivity is superior to the upper limit of the abovementioned range [20].

As regards Relative Enamel Abrasivity (REA), it describes the abrasive potential of a toothpaste on dental enamel. To determine the REA of a toothpaste, the same method and the same standard abrasive is used as in RDA. However, it has been shown that toothpaste RDA could not predict their REA values [21].

It could be hypothesized that the RDA value of pretreating agents is able to influence enamel’s roughness, as well as that eventual debris might remain on the enamel’s surface after its pretreatment, thus affecting the bonding strength. Therefore, the aim of this in vitro and clinical double study is to evaluate the effects of different pretreating agents, with different RDA, used before orthodontic bracket bonding.

The in vitro experimental study aims to evaluate the effect of RDA on Shear Bond Strength (SBS) values and Adhesive Remnant Index (ARI) scores. The clinical trial compares the survival rate of brackets bonded after pretreatment with low and high RDA value toothpastes. The three null hypotheses of the present report were: (1) there was no difference in SBS values among different pretreatments with various RDA toothpastes; (2) there was no difference between ARI scores of the pretreatments with various RDA toothpastes; (3) there was no difference in the clinical survival rate of brackets bonded on enamel pretreated with low RDA value toothpastes compared to high RDA value toothpastes.

## 2. Materials and Methods

### 2.1. In Vitro Study

#### 2.1.1. Specimen Preparation

The Unit Internal Review Board (2018–0530) approved this study. For the in vitro study, 220 freshly extracted bovine lower incisors were collected. They had to meet the following criteria: vestibular and lingual enamel integrity, absence of traumatic lesions related to avulsion and absence of caries.

Established alpha = 0.05 and power = 90%, and considering the SBS primary outcome, sample size calculation required 220 total units. An expected value of 12.6 was hypothesized with a standard deviation of 1.5 [16]. The expected mean difference was supposed to be 2.2. Therefore, 10 samples were required for each group.

After the extraction, teeth were stored in a solution of thymol 0.1% (*w*/*v*) for a week, at 4 °C temperature [22,23]. Initially, periodontal ligament and gingival tissues was cleaned from each tooth with the help of a scalpel; then, they were embedded into cold-curing fast-setting acrylic (Leocryl, Leone s.p.a., Sesto Fiorentino, Italy) inside a plastic cylindrical mold (2 cm height × 2 cm diameter) [24].

Teeth were randomly divided into 22 groups of 10 elements each in order to be subjected to a 1-min pretreatment with different toothpastes and polishing compounds (Table 1). They showed similar values of fluoride (from 1100 to 1450 ppm) so that this variable could not influence the outcome of the study.

In the following section, the pretreating agents used are ordered according to growing values of Relative Dentin Abrasivity (RDA), starting from the commercially available product with the lowest RDA.

- Group 1: cleansing of teeth with fluoride free sodium bicarbonate (Straight Baking Soda, Church and White, Ewing, NY, USA)—RDA = 7.
- Group 2: cleansing of teeth with Super Polish (KerrHawe SA, Bioggio, Switzerland)—RDA = 9.8.
- Group 3: cleansing of teeth with Biosmalto Denti Sensibili (Curaden Helthcare, Varese, Italy)—RDA = 20.
- Group 4: cleansing of teeth with a mixture of water and AIR-FLOW Perio^®^ powder (EMS Electro Medical Systems S.A., Nyon, Switzerland) containing glycine—RDA = 25.
- Group 5: cleansing of teeth with Elmex Sensitive Plus with amine fluoride 1400 ppm (Colgate-Palmolive, New York, NY, USA)—RDA 30.
- Group 6: cleansing of teeth with Advance White Past Baking Soda and Peroxide with sodium fluoride 0.24%-1100 ppm (Church and White, Ewing, NY, USA)—RDA = 42.
- Group 7: cleansing of teeth with CleanPolish (KerrHawe SA, Bioggio, Switzerland)—RDA = 43.8.
- Group 8: cleansing of teeth with a mixture of water and pumice without fluoride (Amedeo Pettinati, Capaccio, Italy)—RDA = 50.
- Group 9: cleansing of teeth with Peroxi Care Regular with sodium fluoride 0.24%-1100 ppm (Church and White, Ewing, NY, USA)—RDA = 52.
- Group 10: cleansing of teeth with Zendium Complete Protection with sodium fluoride 0.24%-1450 ppm (Unilever, Wirral, UK)—RDA = 64.
- Group 11: cleansing of teeth with Colgate Total with sodium fluoride 0.32%-1450 ppm (Colgate-Palmolive, New York, NY, USA)—RDA = 70.
- Group 12: cleansing of teeth with Aim Multi Benefit with sodium fluoride 0.24%-1100 ppm (Church and White, Ewing, NY, USA)—RDA = 80.
- Group 13: cleansing of teeth with Aquafresh Sensitive with sodium fluoride 0.24%-1100 ppm (GSK, Warren, NY, USA)—RDA = 91.
- Group 14: cleansing of teeth with Advance White with sodium fluoride 0.24%-1100 ppm (Church and White, Ewing, NY, USA)—RDA = 106.
- Group 15: cleansing of teeth with Colgate Herbal with sodium fluoride 0.32%-1450 ppm (Colgate-Palmolive, New York, NY, USA)—RDA = 110.
- Group 16: cleansing of teeth with Colgate Whitening with sodium fluoride 0.32%-1450 ppm (Colgate-Palmolive, New York, NY, USA)—RDA = 124.
- Group 17: cleansing of teeth with Crest Extra Whitening with sodium fluoride 0.243%-1100 ppm (P&G, Cincinnati, OH, USA)—RDA = 130.
- Group 18: cleansing of teeth with Ultra Brite Advanced Whitening with sodium fluoride 0.24%-1100 ppm (Colgate-Palmolive, New York, NY, USA)—RDA = 145.
- Group 19: cleansing of teeth with Pespsodent Complete Care with sodium fluoride 0.24%-1100 ppm (Church and White, Ewing, NY, USA)—RDA = 150.
- Group 20: cleansing of teeth with Colgate Tartar Control with sodium fluoride 0.32% -1450 ppm (Colgate-Palmolive, New York, NY, USA)—RDA = 165.
- Group 21: cleansing of teeth with Colgate 2 in 1 Tartar Control/Icy Blast Whitening with sodium fluoride 0.24%-1100 ppm (Colgate-Palmolive, New York, NY, USA)—RDA = 200.
- Group 22—control: teeth from this group were not pretreated with toothpastes before orthodontic bonding, but they were just brushed with water for 1 min.

Groups 2, 7 and 8 were pretreated with the corresponding polishing paste with a toothbrush mounted on a low-speed handpiece for 1 min; successively, the enamel surface was rinsed for 3 s, with the aim of removing residues [25]. Group 4 was treated with a specific handpiece (KaVo Dental GmbH, Biberach an der Riß, Germany) for the use of glycine and bicarbonate; then, all teeth were rinsed for 30 s.

Groups 1, 3, 5, 6, 9, 10, 11, 12, 13, 14, 15, 16, 17, 18, 19, 20 and 21 were pretreated with toothpastes with different RDA values for 1 min, using a medium-bristled toothbrush for home oral care, to simulate manual cleansing before orthodontic bonding; then, all the incisors were rinsed for 20 s [25].

Group 22 was not treated and served as control.

A total of 220 0.022” SWM (Sweden & Martina S.p.A., Due Carrare, Padua, Italy) stainless steel brackets were applied on the vestibular surfaces of teeth following a common protocol for bonding [26]: the vestibular surface of the teeth was etched for 30 s with 37% orthophosphoric acid (Gerhò Etchant gel 37%, Gerhò spa, Terlano, Italy), and then it was rinsed and dried; a thin layer of Transbond XT Light Cure Adhesive Primer (3M Unitek, Monrovia, CA, USA) was applied and then cured for 10 s with a LED curing unit (Starlight Pro, Mectron s.p.a., Carasco, Italy); the bonding of the orthodontic brackets on teeth surfaces was performed with the application of Transbond XT Light Cure Adhesive Paste (3M Unitek, Monrovia, CA, USA) on their base; brackets were applied on the vestibular surfaces with a light pressure to allow the squeezing of the composite from the bracket base [27]. Then, they were correctly oriented and extra paste was removed with a probe; the adhesive paste was cured with the LED curing unit at a distance of 2 mm from the enamel-bracket interface, 10 s on the mesial surface and 10 s on the distal one. Finally, specimens were stored in a thymol solution 0.1% (*w*/*v*) at room temperature [28]. The characteristics of the materials used and the protocols recommended for their application are shown in Table 2.

#### 2.1.2. Shear Bond Strength (SBS) Test

SBS was evaluated for each tooth using a universal testing machine (Model 3343, Instron, Canton, MA, USA) [29]. Specimens were positioned in the lower jaw of the machine, at the exact center of their mold and inserted in a way that the shear force is exerted parallel to the bases of the brackets. The load was exerted in an occlusal-gingival direction, and the blade of the machine was set at 1 mm/min speed [30]. The maximum load necessary for the detachment was registered in Newton, using the software Bluehill 2 (Instron Industrial Products, Grove City, PA, USA). Data were converted into megapascals, knowing the area of the bracket’s base (MPa = N/mm^2^).

#### 2.1.3. Adhesive Remnant Index (ARI) Score

The bases of the brackets and the surfaces of bonding were analyzed at X 10 magnification with a microscope (Stereomicroscope SR, Zeiss, Oberkochen, Germany), in order to determine the remaining adhesive after the detachment; therefore, ARI [30,31] was calculated with the following scoring criteria: 0: no adhesive; 1: less than 50% of adhesive remaining; 2: more than 50% of adhesive remaining; and 3: 100% of adhesive left.

### 2.2. Randomized Clinical Trial (RCT)

#### 2.2.1. Trial Design

The Unit Internal Review Board (2019–0403) approved the study. It was a parallel group, randomized, active controlled, split-mouth and single-center trial with a 1:1 allocation ratio.

#### 2.2.2. Sample Size Calculation

Sample size calculation (alpha 0.05; power = 85%) for two independent study groups and a dichotomous primary endpoint was performed. Concerning the variable “failure rates”, an expected value of 2.08% was hypothesized. The expected difference between the percentages was supposed to be 6.78% [17]; therefore, 200 brackets per group were requested. Considering 20 brackets bonded for each patient, with a split-mouth design, 20 patients were necessary for each group.

Interim analysis and stopping guidelines were not applicable.

#### 2.2.3. Participants

For this study, patients were enrolled from the Unit of Orthodontics and Pediatric Dentistry, Section of Dentistry, Department of Clinical, Surgical, Diagnostic and Pediatric Sciences, University of Pavia, Pavia, Italy. Recruitment started in June 2019 and the study ended in March 2021. Informed consent was obtained for each participant; for underage patients, the consent was signed by their parents. The inclusion criteria were patients scheduled for brackets’ placement at least for one dental arch; only permanent teeth were considered for bonding. The exclusion criteria were teeth with bands placed, teeth with restorations, teeth with prosthetic crown and teeth with direct tubes.

#### 2.2.4. Intervention

The aim of the clinical study was to investigate the failure rate of orthodontic brackets after a pretreating procedure with two different toothpastes. The split-mouth design enabled the possibility of using the same patient for the administration of two different treatments.

Patients from group A received enamel pretreatment with a low RDA toothpaste (Advance White Paste Baking Soda and Peroxide, Church and White, Ewing, NY, USA; RDA = 42) in the maxillary left and mandibular right quadrants, while the remaining quadrants were pretreated with a high RDA toothpaste (Colgate 2 in 1 Tartar Control/Icy Blast Whitening, Colgate-Palmolive, New York, NY, USA; RDA = 200). In group B, the quadrants were inverted. The sides were allocated using random number tables.

In Table 3 are shown the compositions of the two toothpastes; since fluoride can influence the curing procedure and alter bonding efficacy, two toothpastes had been chosen with the same fluoride content.

Successively, 400 0.022” 3M (3M Unitek, Monrovia, CA, USA) stainless steel brackets were bonded on the vestibular surfaces of teeth. These surfaces were etched for 30 s with 37% orthophosphoric acid (Gerhò Etchant gel 37%, Gerhò spa, Terlano, Italy), then rinsed and dried; a thin layer of Transbond XT Light Cure Adhesive Primer (3M Unitek, Monrovia, CA, USA) was applied and then cured for 10 s with a LED curing unit (Starlight Pro, Mectron s.p.a., Carasco, Italy); the bonding of the orthodontic brackets on teeth surfaces was performed with the application of Transbond XT Light Cure Adhesive Paste (3M Unitek, Monrovia, CA, USA) on their bases; then, brackets were applied on the vestibular surfaces with a light pressure and correctly oriented, and extra paste was removed with a probe; the adhesive paste was cured with the LED curing unit at a distance of 2 mm from the enamel-bracket interface, 10 s on the mesial surface and 10 s on the distal one.

#### 2.2.5. Outcomes

Data collection was carried out during monthly visits in a time period between 1 month and 12 months from the day of brackets’ placement. Each detachment was registered distinguishing the specific tooth interested and assessing the time period from the initial bonding procedure. The teeth with the detached brackets were not further included in the study. Participants were reminded to attend the appointments and to immediately inform the orthodontist if any detachment had happened. No variation to the outcome occurred after the beginning of the trial.

#### 2.2.6. Randomization and Blinding

A randomization sequence was generated by the data analyst thanks to a block randomization table; a permuted block of 20 participants was considered. Participants were allocated by the operator, who enrolled them using sequentially numbered and sealed envelopes with the allocation cards previously prepared. The operator, participants, data assessor and data analyst were always blinded during the study and none of them knew which treatment toothpaste had been used. Patients could not note any differences except from the two tastes of the toothpastes.

#### 2.2.7. Allocation Concealment

The operator who enrolled participants also achieved the allocation concealment using sequentially numbered and sealed envelopes containing allocation cards that had previously prepared. The randomization list generated was held securely in remote location.

### 2.3. Statistical Methods

Data analysis was conducted with R software (R version 3.1.3, R Development Core Team, R Foundation for Statistical Computing, Wien, Austria). For SBS values, descriptive statistics were calculated for each of the twenty-two groups. Data included mean, standard deviation, minimum, median and maximum SBS values. The Kolmogorov–Smirnov test was used for calculating data normality. Successively, an ANOVA test was performed, followed by Tukey’s test for post-hoc analysis. A Pearson correlation coefficient was calculated to determine whether there was any correlation between SBS and RDA values.

For the ARI score, a χ^2^ test was conducted to assess significant differences between the twenty-two groups. A Spearman correlation coefficient was calculated to determine any correlation between ARI and RDA values.

The aim of the RCT was assessed with a Fisher exact test to determine differences among the frequencies of brackets’ detachments of the two experimental groups; finally, Kaplan-Meier survival curves of group 1 and 2 were constructed and compared using the log-rank test.

Significance for all statistical tests was predetermined at *p* < 0.05.

## 3. Results

### 3.1. In Vitro Test

#### 3.1.1. Shear Bond Strength (SBS) Test

**Figure 1:**
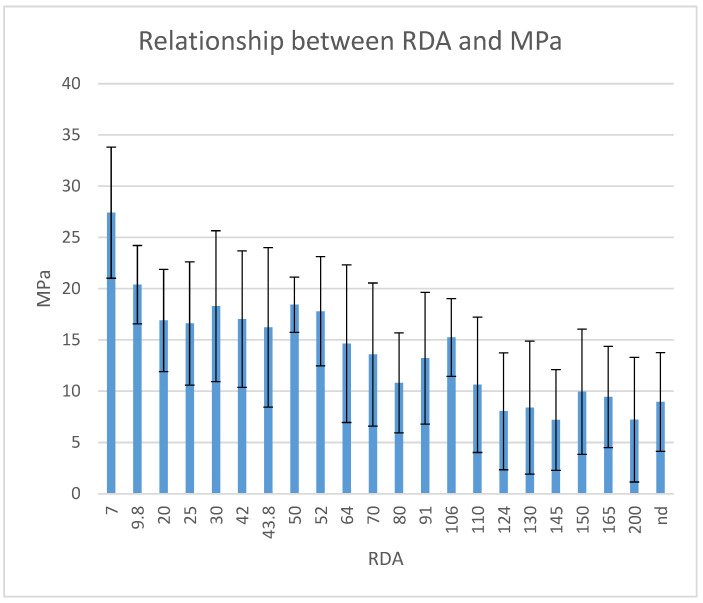
In vitro SBS values (in MPa) for each agent tested.

Descriptive statistics for the twenty-two groups are shown in Table 4 and Figure 1. The ANOVA test showed significant differences between groups (*p* < 0.0001). Tukey’s post-hoc test revealed that teeth without pretreatment or treated with toothpastes with high RDA values (Colgate Whitening—124; Crest Extra Whitening–130; Ultra Brite Advanced Whitening-145; Pepsodent Complete Care—150; Colgate Tartar Control—165; Colgate 2 in 1 Tartar Control/Icy Blast Whitening—200) did not have significantly different SBS values (*p* > 0.05); SBS values were significantly lower if compared to the other groups (*p* < 0.05). The Pearson correlation coefficient showed a moderate correlation between SBS and RDA values (r = −0.5779).

#### 3.1.2. Adhesive Remnant Index (ARI)

Descriptive statistics for the twenty-two groups are shown in Table 5. The χ^2^ test showed that there is a statistically significant higher frequency of ARI scores for 2 and 3 of the twenty-two groups (*p* < 0.05). Spearman coefficient showed a weak correlation between ARI and RDA values (r = 0.2467).

### 3.2. Randomized Clinical Trial (RCT)

**Figure 2:**
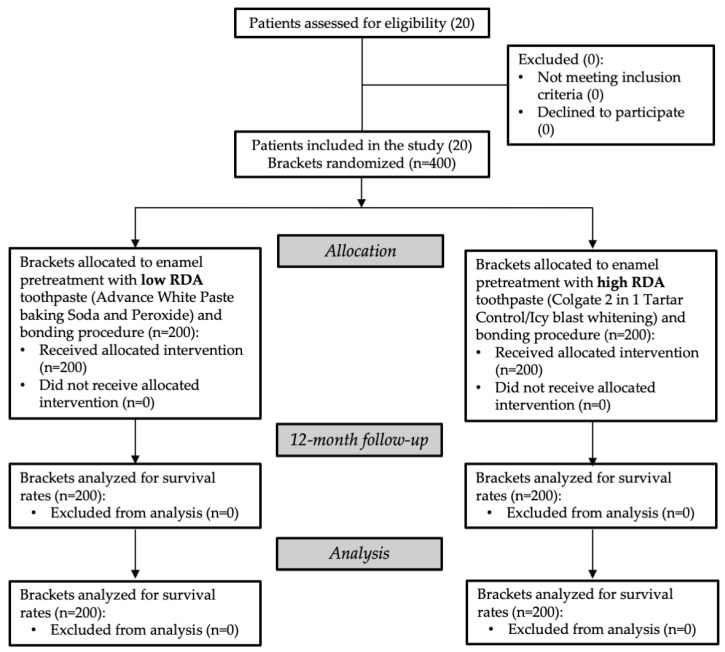
Flow chart showing participants and protocol used in this study.

A total of 20 patients (13 females, mean age 17 years and 8 months; 7 males, mean age 17 years and 6 months) were enrolled for the study as they agreed with the inclusion criteria. They all received allocated intervention and none of them were excluded from analysis. The flow chart of the study is shown in Figure 2.

As shown in Table 6, statistically significant differences in the cumulative failure rate were found between the two groups (*p* < 0.05).

No statistically significant difference was found between anterior and posterior sites (Table 7) and between the two dental arches (Table 8).

**Figure 3:**
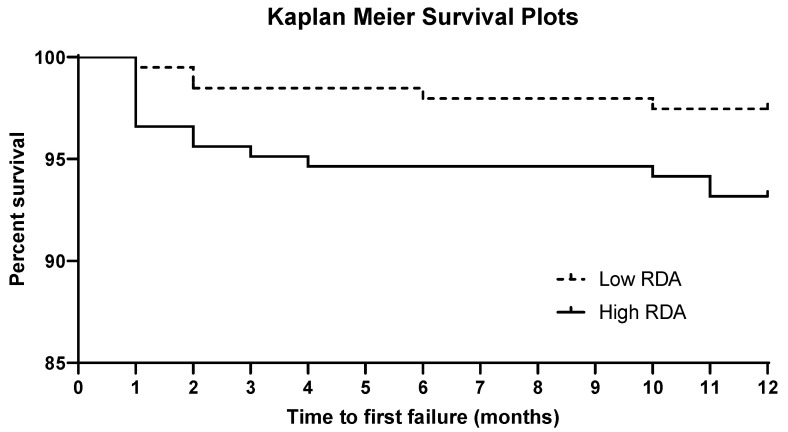
Kaplan-Meier survival plots for the two groups.

Kaplan-Meier Survival curves for low and high RDA values groups are illustrated in Figure 3. During the time frame of the study, a statistically significant difference was found between the two groups (hazard ratio: 0.34; C.I. 95%: 0.15–0.92; log rank test: *p* = 0.0315).

## 4. Discussion

Brackets’ detachment is a widely investigated issue, with an extensive literature of in vitro and clinical studies [8,10,12,13,14,23]. The aim of the present study was to test different pretreating agents to find eventual differences among the products in order to assess which could cause lower detachment rate.

The first null hypothesis of the present report has been rejected because there were significant differences among the groups as regards SBS values. The main variable considered is have SBS and higher SBS values been found among in vitro studies [32] due to the ideal conditions in which they are conducted; surely, the absence of a wet environment is a significant contributing factor in the adhesion process [21]. However, in vitro experimentations are useful to predict the behavior of materials tested when used in the oral environment.

In the adhesion process, apart from the polymeric adhesive material used [7], the material of the brackets [8] and the direct or indirect bonding procedure adopted [23], enamel etching with 37% orthophosphoric acid is the first step in order to achieve a performing bonding procedure. This because it creates an uneven surface topography and the opening of the interprismatic areas, conditions that allow the mechanical retention of the polymeric adhesive applied on the brackets’ base [33]. However, in literature different pretreating agents have been considered before the acid etching procedure: casein phosphopeptide and/or amorphous calcium phosphate (CPP-APC) [15,16,34,35,36], fluoride varnishes/gels [15,16,35,36], air abrasion with aluminium oxide [9,37], hydroxyapatite and glycine [15], ozone [15,38], fluoridated and non-fluoridated prophylactic pastes [39], 5.2% NaOCl [9,40], laser abrasion [37], resin infiltrant [16,36] and desensitizers and bleaching gels [37].

The rationale of the present study, which involved both in vitro and clinical phases, was to assess if there could be a role of enamel pretreatment in the failure rates of brackets bonding, when toothpastes with different RDA values are used before the acid etching procedure.

The use of bovine teeth is a limitation of this study. However, despite shape and size differences, they show similar physical properties in regard to human teeth, therefore, they can be used instead of them for in vitro experimentations [41].

The in vitro part of the present study showed that higher RDA values correspond to significantly lower SBS values, and this trend was confirmed by the Pearson coefficient, which showed a moderate negative correlation between SBS and RDA values (r = −0.5779). This means that only 33.3% of the variance of SBS is explained by the RDA. Due to the fact that no previous similar studies have been conducted, it is not possible to compare the Pearson correlation coefficients.

The control group showed SBS values similar to those of other in vitro studies that used Transbond XT as the adhesive system with a similar protocol [15,34,37]; Huilcapi and colleagues [9], instead, used a different adhesive system, but the results are still comparable.

The study of Cossellu and colleagues [15] is the only one that used glycine and a toothpaste in enamel pretreatment, as in the present work. Additionally, also fluoride varnish, casein-phosphopeptide-amorphous calcium-phosphate (CPP_ACP), ozone and hydroxyapatite powder were tested. These three substances did not compromise on bracket bond strength. Conversely, fluoride, glycine and hydroxyapatite significantly decreased the SBS. Anyway, only the fluoride group showed significant clinically low (<6 MPa) SBS values.

In detail, Air-Flow Perio containing glycine was used with the same protocol as for group 4 of the present study, together with the adhesive technique used; SBS values are comparable (11.02 MPa vs. 12.44 MPa, respectively). The toothpaste used is Biorepair Plus (RDA ~70), which has similar RDA to Colgate Total (RDA = 70) toothpaste used in group 11 (16.01 MPa vs. 11.63 MPa, respectively). However, it should be highlighted that the latter contains fluoride (1450 ppm), while the former does not. The control groups show a slight difference between SBS values (17.38 MPa vs. 11.01 MPa).

The study of Mahajan and colleagues [39], instead, used flour of pumice similarly to the present work for group 8, but its RDA value is unknown, and a Self-Etch Primer was used for brackets’ bonding, therefore, a proper comparison is not suitable.

Enamel pretreatment before acid etching could improve adhesion values, because its purpose is to remove the biofilm present on the surface of teeth and allow a better adhesion of the bracket [40]; however, the strength of adhesion to the teeth is highly influenced by its degree of demineralization [26]. According to a recent systematic review, the incidence of white spot lesions in orthodontic patients is widely variable [42] and different solutions have been proposed, concluding that a fluoride toothpaste is the best treatment option in absence of evidence-based findings. The results of fluoride application before, during and after the acid-etching procedure are controversial [43]; nevertheless, the in vitro study tested pretreating agents with almost the same quantity of fluoride, with the aim of limiting its effects on the bonding procedures.

All SBS values are included within 5–50 MPa, which is considered a clinically acceptable theoretic range for orthodontic biomaterials [30].

The second null hypothesis of the present study was rejected because there were significant differences among the groups as regards ARI scores. In fact, there was a statistically significant higher frequency of ARI scores for 2 and 3 in the 22 groups of the in vitro study. The Spearman correlation coefficient showed a weak correlation between ARI and RDA values. According to a previous report in which powders and toothpastes were compared [15], lower scores of ARI have been found for both the pretreatment agents, while in the present work, higher ARI scores were found. The same scenario occurred for the control groups. Moreover, it was not possible to consider a comparison with the work of Mahajan and colleagues for the flour of pumice [39] because ARI evaluation was not performed.

ARI is one of the most used methods for the evaluation of adhesive systems for the bonding of orthodontic brackets [44]. A score of 0 is related to low SBS values and to contaminants over enamel; a score of 3, instead, means less risk of enamel fracture after debonding, but the removal of the remnant adhesive on enamel surfaces is longer and should be done carefully [30].

Finally, also the third null hypothesis was rejected. When enamel was pretreated with low RDA toothpaste, lower failure rates were reported.

Toothpastes are widely used for routine home oral care [45], and evaluation of their values of abrasion with reference to enamel and dentin (Relative Dentin Abrasion—RDA—and Relative Enamel Abrasion—REA—values) should be taken into consideration in the choice of a toothpaste, considering that they could play a role as pretreating agents. However, not all toothpastes show REA values [19], therefore, RDA is one of the parameters that deserves to be investigated as a potential modifier of the traditional bonding procedure of orthodontic brackets.

Various clinical studies tried to estimate the failure rate of orthodontic brackets, comparing brackets of different materials [8], types of orthodontic bonding [23], adhesion procedures [46] and adhesive materials [7,17,47]. In addition, retrospective studies investigated the issue, trying to assess correlations between brackets’ failure rate and multiple variables [11,12,48].

To date, there is no clinical study that has evaluated the efficacy of toothpastes as pretreating agents. Generally, the most used pretreatment agent is pumice together with water, with a rubber cup mounted on a low-speed handpiece. The study of Burgess and colleagues [49] evaluated the effect of pumice and water as enamel pretreating agents on brackets’ adhesion, using a rubber cup, reporting a significant difference between pumiced and non-pumiced groups, as brackets placed on pumiced teeth showed lower failure rates. Other clinical studies considered enamel pretreatment with a rubber cup, pumice and water for metallic brackets bonding.

Krishnan et al. [47] adopted the same adhesion protocol and materials as in the present study, showing a failure rate of 8.1%. For the other group, in which a flowable resin composite was used (Heliosit Orthodontic, Ivoclar Vivadent AG, Schaan, Liechtenstein), a lower failure rate (6%) was reported. The study of Dominguez et al. [46] compared two different adhesion protocols: 37% etching + Transbond XT paste and Transbond Self Etch Primer + Transbond XT paste (3M Unitek, Monrovia, CA, USA) with previous pumicing with a rubber cup. The survival rates for the two groups were, respectively, 5.41% and 4.58%.

Ogiński et al. [8] found a 7.2% failure rate for 100 stainless steel brackets in a twelve-month study, but even though the same adhesive material was used (Transbond SEP + XT, 3M Unitek, Monrovia, CA, USA), neither pretreatment nor etching with 37% orthophosphoric acid were executed. Kafle et al. [12] found a failure rate of 3.34% for metallic brackets, but information about materials and protocol used are missing.

Among all these pretreating agents used, further analyses are required to understand what happens to pretreated enamel. As an example, Ravichandran et al. [49] used optical coherence tomography to assess in vitro enamel loss after pumicing and etching. The findings of this work highlight that the combination of the two procedures increases enamel loss with a slight difference between etching only. However, it should be evaluated if possible residual remnants of pumice lie on the enamel surface, thus hindering the adhesion procedure. Moreover, other evaluations should be performed, with other pretreating agents, to assess the changes on the enamel surface after the procedure.

The lack of standardized procedures for the bonding of orthodontics brackets in the abovementioned clinical studies leads to a variety of different materials used and protocols adopted. For this reason, a direct comparison among the failure rates of brackets bonded on pretreated enamel is not possible. However, an analysis of the failure rates could help in determining the possible role of pretreatment procedures in the adhesion process.

The failure rates of the previous studies, including the present research, are within the range of clinical acceptability of 10% [12], except from the study of Burgess et al. [50]. In detail, the failure rate of the group pretreated with the low RDA toothpaste (2.5%) is the lower value among the abovementioned ones, although different protocols were used; the survival rate of the trial group with high RDA toothpaste pretreatment is similar to other previous reports (7%). Since the RDA values of pumice flours used is unknown, together with the time of execution, a clear role of the pretreatment procedure is uncertain. However, in this study, a statistically significant difference has been found between the two groups. Other variables should be considered in the assessment of failure rates causes. For example, Sukhia et al. [48] developed a multivariate estimated model with different factors that could affect the bonding failure rate, including bracket material, jaw (maxilla/mandible), overjet, overbite, site (anterior/posterior), side (left/right) and the interaction between site and side. The results highlighted that in the posterior region the risk is considerably higher if compared to the anterior region; also, mandibular brackets were more prone to fail if compared to the maxillary ones.

In particular, the risk of bracket failure on the right posterior region appears to be 7.7 times that in the right anterior region when adjusted for all other variables in the model (HR: 7.7; 95% CI: 4.3–13.6).

Stasinopoulos et al. [11] considered linear bi- and multivariate regression; they concluded that posterior brackets were more prone to failure. Specifically, considerable differences existed according to tooth type, with an 8.0% failure of canines, followed by 11.3% for first premolars, 13.1% for central incisors, 14.4% for lateral incisors and 23.4% for second premolars.

These data are partially in agreement with the present report, as failure rates were higher in the lower and posterior sites but with no significant difference. Additionally, the results of the present report agree with another study that tested orthodontic splints using the same polymeric composite resin and that reported no significant difference between upper and lower frameworks [28].

Due to the absence of previous similar randomized clinical trials on pretreating agents in orthodontics, further studies are desired to compare the results obtained here. Additionally, one of the limitations of the present study is that the toothpastes are not directly comparable because they differ not only in terms of their RDA but also their composition. In addition to that, the fluoride level cannot be completely controlled because manufacturers commercialize their products with inconstant fluoride percentages. Another concern in the clinical study is that the malocclusion type was not taken into consideration. Therefore, future studies testing other variables and with longer follow-up are expected to clarify the best enamel pretreatment protocol before bracket bonding. Moreover, it could be hypothesized that the increase of the amount of debris left on the enamel after the pretreatment procedure, along with its increasing roughness, are responsible for the low SBS values found in the case of using high RDA agents [51]. Since it was not the purpose of this study, it would be interesting to perform additional microscopical and chemical evaluations, respectively with SEM (Scanning Electron Microscopy) and EDS (Energy Dispersive X-ray Spectroscopy) analysis, in order to better understand how pretreating agents affect enamel’s morphology and composition. Moreover, the different bristles’ hardness of the rubber cup used for the pretreatment procedure might have an influence on the parameters tested, which should be assessed. In fact, despite RDA being a standardized and reasonably valid tool to determine the abrasive power of toothpastes, behavioral differences among individuals significantly influence the potential of abrasion of a specific agent, independently of its RDA value [52].

Due to the high number of commercial materials tested in this study, further variables, like the presence of different excipients in the products, should be taken into account. Finally, other pretreatment methods (like airflow) deserve to be tested and compared with respect to the treatments considered in the present study.

## 5. Conclusions

Enamel pretreatment before standard adhesion protocols for orthodontic brackets with low RDA toothpastes could be more suitable than those with higher RDA values as their bonding survival rate is significantly higher.

## Figures and Tables

**Table 1:** Materials used and their composition.

Product	Manufacturer	RDA	Fluoride	F (ppm)	Composition
Straight Baking Soda	Church and Dwight (Ewing, NY, USA)	7	-	-	100% sodium bicarbonate
Super Polish	KerrHawe SA (Bioggio, Switzerland)	9.8	-	-	Humectants, abrasive (alumina), binders, flavoring substances (anethole, mint), methylparaben, coloring substances (CI 14720), gluten free
Biosmalto Denti Sensibili	Curaden Healthcare (Varese, Italy)	20	Fluoro-hydroxyapatite	1450	Purified water, glycerin, hydrated silica, strontium chloride, fluorapatite, chitosan-combined Mg-Sr-carbonate hydroxyapatite, cellulose gum, xylitol, cocamidopropyl betaine, xanthan gum, potassium acesulfate, flavor, phenoxyethanol, sodium benzoate, citric acid.
Glycine	KaVo Dental GmbH (Biberach an der Riß, Germany)	25		-	C_2_H_5_NO_2_, water, magnesium, calcium, zinc
Elmex Sensitive Plus	Colgate-Palmolive (New York, NY, USA)	30	Amine fluoride	1400	Water, sorbitol, glycerin, polyethylene, ethylene, hydrated silica, hydroxyethyl cellulose, flavor, silica, dimethyl silicate, olaflur, sodium saccharin, CI 77891
Advance White Paste Baking Soda and Peroxide	Church and Dwight (Ewing, NY, USA)	42	Sodium fluoride (0.243%)	1100	Sodium bicarbonate, PEG-8, tetrasodium pyrophosphate, PEG-PPG 116/66 copolymer, sodium carbonate peroxide, silica, sodium saccharin, flavor, sodium sarcosinate, water, sodium lauryl sulfate
Clean Polish	KerrHawe SA (Bioggio, Switzerland)	43.8	-	-	Humectants, abrasive (pumice, alumina, calcium, carbonate), binders, flavoring substances (anethole, mint), methylparaben, coloring substances (CI14720), gluten free
Pumice	Amedeo Pettinati (Capaccio, Italy)	50	-	-	Silica, aluminium oxide, ferric oxide, magnesium oxide, calcium oxide, sodium oxide, potassium oxide, manganese oxide, titanium oxide
Peroxi Care Regular	Church and Dwight (Ewing, NY, USA)	52	Sodium fluoride (0.24%)	1100	Sodium bicarbonate (baking soda), PEG-8, PEG-PPG 116/66 copolymer, sodium carbonate peroxide, tetrasodium pyrophosphate, silica, sodium saccharin, flavor, water, sodium lauroyl sulfate, sodium lauroyl sarcosinate
Zendium Complete Protection	Unilever (Wirral, UK)	64	Sodium fluoride (0.24%)	1450	Water, hydrated silica, sorbitol, glycerin, steareth-30, xanthan gum, flavor, carrageenan, disodium phosphate, sodium fluoride, amyloglucosidase, citric acid, zinc gluconate, sodium benzoate, glucose oxidase, sodium saccharin, potassium thiocyanate, lysozyme, colostrum, lactoferrin, lactoperoxidase, CI 77891
Colgate Total	Colgate-Palmolive (New York, NY, USA)	70	Sodium fluoride (0.32%)	1450	Water, hydrated silica, glycerin, sorbitol PVM/MA copolymer, sodium lauryl sulfate, flavor, cellulose gum, sodium hydroxide, propylene glycol, carrageenan, sodium saccharin, titanium dioxide
Aim Multi Benefit	Church and Dwight (Ewing, NY, USA)	80	Sodium fluoride (0.24%)	1100	Sorbitol, water, hydrated silica, PEG-8, sodium lauryl sulfate, SD alcohol 38-B, flavor, cellulose gum, sodium saccharin, titanium dioxide
Aquafresh Sensitive	GSK (Warren, NY, USA)	91	Sodium fluoride (0.24%)	1100	D&C RED NO. 30, FD&C BLUE NO. 1, aluminium oxide, glycerin, hydrated silica, sodium benzoate, sodium hydroxide, sodium lauryl sulfate, sodium saccharin, sorbitol, titanium dioxide, water, xanthan gum
Advance White	Church and Dwight (Ewing, NY, USA)	106	Sodium fluoride (0.24%)	1100	Sorbitol, sodium bicarbonate, water, hydrated silica, glycerin, sodium pyrophosphate, sodium lauryl sulfate, saccharin sodium, sodium lauryl sarcosinate, carboxymethyl cellulose sodium, FD&C BLUE NO. 1, FD&C YELLOW NO. 5
Colgate Herbal	Colgate-Palmolive (New York, NY, USA)	110	Sodium fluoride (0.32%)	1450	Water, glycerin, hydrated silica, sodium lauryl sulfate, cellulose gum, flavor, sodium fluoride, sodium saccharin, commiphora myrrha oil, chamomilla, recutita flower extract, salvia officinalis oil, mentha piperita oil, eucalyptus globulus leaf oil, limonene, CI 777891, CI 74260
Colgate Whitening	Colgate-Palmolive (New York, NY, USA)	124	Sodium fluoride (0.32%)	1450	Calcium carbonate, water, sorbitol, sodium lauryl sulfate, hydrated silica, flavor, sodium monofluorophosphate, cellulose gum, magnesium aluminium silicate, sodium carbonate, benzyl alcohol, sodium saccharin, sodium bicarbonate, cinnamal, eugenol, limonene
Crest Extra Whitening	P&G (Cincinnati, OH, USA)	130	Sodium fluoride (0.243%)	1100	Sorbitol, water, hydrated silica, sodium acid pyrophosphate, sodium lauryl sulfate, sodium hydroxide xanthan gum, saccharin sodium, carnauba wax, titanium dioxide, FD&C BLUE NO. 1, FD&C YELLOW NO. 5
Ultra Brite advanced whitening	Colgate-Palmolive (New York, NY, USA)	145	Sodium fluoride (0.24%)	1100	Sorbitol, water, hydrated silica, PEG-12, sodium lauryl sulfate, flavor, cellulose gum, tetrasodium pytophosphate, cocamidepropyl betaine, sodium saccharin, titanium dioxide
Pepsodent Complete Care	Church and Dwight (Ewing, NY, USA)	150	Sodium fluoride (0.24%)	1100	Sorbitol, water, hydrated silica, PEG-8, sodium lauryl sulfate, SD alcohol 38-B, flavor, cellulose gum, sodium saccharin, titanium dioxide
Colgate Tartar Control	Colgate-Palmolive (New York, NY, USA)	165	Sodium fluoride (0.32%)	1450	Water, hydrated silica, sorbitol, glycerin, PEG-12, pentasodium triphosphate, tetrasodium pyrophosphate, sodium lauryl sulfate, flavor, cellulose gum, sodium fluoride, carrageenan, sodium saccharin, limonene, CI 77891
Colgate 2 in 1 Tartar Control/Icy blast whitening	Colgate-Palmolive (New York, NY, USA)	200	Sodium fluoride (0.24%)	1100	Water, sorbitol, hydrated silica, glycerin, sodium lauryl sulfate, flavor, tetrasodium saccharin, cocamidopropyl betaine, cellulose gum, xanthan gum, titanium diocide

**Table 2:** Characteristics of the materials used, and application protocols recommended by the manufacturers.

Material	Type	Composition	Application Protocol
Gerhò Etchant gel 37%	Etchant gel	Orthophosphoric acid (37%)	Teeth polishing with a pretreating agent Rinsing Tooth isolation with cotton rolls Etching for 30 s Thorough rinsing with water and air-drying
Transbond XT Primer	Filler-free, light-cured liquid resin	BisGMA, TEGDMA	Application of a thin uniform coat of Primer on the vestibular surface of the tooth to be bonded Air-blowing Photopolymerization (10 s)
Transbond XT Light Cure Adhesive Paste	Filler-reinforced, light-cured paste	Silane-treated quartz (70–80%), bis-GMA (10–20%), bisphenol A bis(2-hydroxyethyl ether) dimethacrylate (5–10%), silane-treated silica (<2%), DPIHFP (<0.2%)	Application on the base of the brackets. Photopolymerization for 20 s

**Legend**: bis-GMA, bisphenol A diglycidyl ether dimethacrylate; TEGDMA, triethylene glycol dimethacrylate; DPIHFP, diphenyliodonium hexafluorophosphate.

**Table 3:** Composition of the two toothpastes used for RCT.

Toothpaste	Manufacturer	Fluoride	F (ppm)	RDA	Composition
Advance White Paste Baking Soda and Peroxide	(Church and White, Ewing, NJ, USA)	Sodium fluoride 0.24%	1100	42	Sodium bicarbonate, PEG-8, tetrasodium pyrophosphate, PEG-PPG 116/66 copolymer, sodium saccharin, silica, flavor, sodium sarcosinate, water, sodium lauryl sulfate
Colgate 2 in 1 Tartar Control/Icy Blast Whitening	(Colgate-Palmolive, New York, NY, USA)	Sodium fluoride 0.24%	1100	200	Sorbitol, hydrated silica, glycerin, sodium lauryl sulfate, flavor, tetrasodium saccharin, cocamidroproyl betaine, cellulose gum, water, xanthan gum, titanium diocide.

**Table 4:** RDA and SBS values.

Toothpaste (Commercial Name)	RDA	SBS Values					
		Mean	SD	Min	Mdn	Max	*
Straight Baking Soda	7	27.42	6.392	18.74	26.86	36.46	A
Super Polish	9.8	20.4	3.82	15.21	20.8	24.95	A,B
Biosmalto Denti Sensibili	20	16.91	4.985	10.6	15.56	24.68	A
Air-Flow Perio (glycine)	25	16.61	6.016	11.61	14.58	28.45	A
Elmex Sensitive Plus	30	18.3	7.357	6.8	20.6	27.34	A
Advance White Paste Baking Soda and Peroxide	42	17.04	6.653	7.27	19.13	23.63	A,B
Polish Verde	43.8	16.24	7.786	2.98	19.25	22.67	A,B
Pumice	50	18.44	2.687	15	17.94	22.78	A,B
Peroxi Care Regular	52	17.8	5.328	13.74	15.1	28.46	A,B
Zendium Complete Protection	64	14.64	7.685	2.49	16.63	23.77	C,B
Colgate Total	70	13.59	6.976	4.55	12.79	24.62	B,C
Aim Multi Benefit	80	10.82	4.873	4.53	10.84	18.14	B,C
Aquafresh Sensitive	91	13.22	6.418	2.76	16.64	19.57	B,C
Advance White	106	15.24	3.785	7.88	14.68	20.28	B,C
Colgate Herbal	110	10.63	6.602	1.29	11.61	19.58	B,C
Colgate Whitening	124	8.05	5.704	1.19	9.48	15.24	C,D
Crest Extra Whitening	130	8.406	6.49	1.52	6.3	16.63	C,D
Ultra Brite Advanced Whitening	145	7.197	4.906	2.14	6.33	13.03	C,D
Pepsodent Complete Care	150	9.952	6.105	3.36	9.21	21.26	B,C
Colgate Tartar Control	165	9.441	4.933	2.32	8.43	18.63	B,C
Colgate 2 in 1 Tartar Control/Icy Blast Whitening	200	7.226	6.073	1.41	4.38	17.15	C,D
Control	nd	8.954	4.814	2.41	9.975	16.48	B,C

* Significance: groups with same letters show no significantly different means.

**Table 5:** RDA and ARI values.

Toothpaste (Commercial Name)	RDA	ARI			
		0 (%)	1 (%)	2 (%)	3 (%)
Straight Baking Soda	7	2 (20)	2 (20)	2 (20)	4 (40)
Super Polish	9.8	3 (30)	1 (10)	3 (30)	3 (40)
Biosmalto Denti Sensibili	20	0 (0)	1 (10)	5 (50)	4 (40)
Air-Flow Perio (glycine)	25	3 (30)	2 (20)	0 (0)	5 (50)
Elmex Sensitive Plus	30	2 (20)	0 (0)	1 (10)	7 (70)
Advance White Paste Baking Soda and Peroxide	42	0 (0)	1 (10)	2 (20)	7 (70)
Polish Verde	43.8	3 (30)	2 (20)	0 (0)	5 (50)
Pumice	50	5 (50)	1 (10)	2 (20)	2 (20)
Peroxi Care Regular	52	0 (0)	1 (10)	0 (0)	9 (90)
Zendium Complete Protection	64	0 (0)	0 (0)	2 (20)	8 (80)
Colgate Total	70	0 (0)	1 (10)	3 (30)	6 (60)
Aim Multi Benefit	80	0 (0)	1 (10)	2 (20)	7 (70)
Aquafresh Sensitive	91	1 (10)	2 (20)	3 (30)	4 (40)
Advance White	106	2 (20)	1 (10)	4 (40)	3 (30)
Colgate Herbal	110	2 (20)	1 (10)	1 (10)	6 (60)
Colgate Whitening	124	0 (0)	1 (10)	0 (0)	9 (90)
Crest Extra Whitening	130	2 (20)	1 (10)	2 (20)	5 (50)
Ultrabrite Advanced Whitening	145	1 (10)	2 (20)	0 (0)	7 (70)
Pepsodent Complete Care	150	0 (0)	1 (10)	0 (0)	9 (90)
Colgate Tartar Control	165	0 (0)	0 (0)	0 (0)	10 (100)
Colgate 2 in 1 Tartar Control/Icy Blast Whitening	200	0 (0)	0 (0)	2 (20)	8 (80)
Control	nd	0 (0)	0 (0)	2 (20)	8 (80)

**Table 6:** Comparison of failure rates between low and high RDA values for the total amount of brackets bonded.

Brackets	n. Bonded	n. Failed	Bond Failure (%)	Significance
Low RDA	200	5	2.50	
High RDA	200	14	7.00	0.0315
Total	400	19	4.75	

**Table 7:** Failure rates of brackets per site (anterior and posterior).

		Anterior			Posterior		
Treatment	n. Bonded	n. Failed	% Failed	n. Bonded	n. Failed	% Failed	Paired *t*-Test
Low RDA	120	3	2.50	80	3	3.75	ns
High RDA	120	5	4.17	80	9	11.25	ns
Total	240	8	3.33	160	12	7.50	ns

**Table 8:** Failure rates of brackets per arch (upper and lower).

	Upper Arch			Lower Arch			
Treatment	n. Bonded	n. Failed	% Failed	n. Bonded	n. Failed	% Failed	Paired *t*-Test
Low RDA	100	3	3.00	100	3	3.00	ns
High RDA	100	4	4.00	100	10	10.00	ns
Total	200	7	3.50	200	13	6.50	ns

## Data Availability

Data are available upon reasonable request to the Corresponding Authors.

## References

[1] De Souza R.A., de Oliveira A.F., Pinheiro S.M., Cardoso J.P., Magnani M.B. (2013). Expectations of orthodontic treatment in adults: The conduct in orthodontist/patient relationship. Dental Press J. Orthod..

[2] Papageorgiou S.N., Koletsi D., Iliadi A., Peltomaki T., Eliades T. (2020). Treatment Outcome with Orthodontic Aligners and Fixed Appliances: A Systematic Review with Meta-Analyses. Eur. J. Orthod..

[3] Ata-Ali F., Plasencia E., Lanuza-Garcia A., Ferrer-Molina M., Melo M., Ata-Ali J. (2019). Effectiveness of lingual versus labial fixed appliances in adults according to the Peer Assessment Rating index. Am. J. Orthod. Dentofac. Orthop..

[4] Baricevic M., Mravak-Stipetic M., Majstorovic M., Baranovic M., Baricevic D., Loncar B. (2011). Oral mucosal lesions during orthodontic treatment. Int. J. Paediatr. Dent..

[5] Dowsing P., Murray A., Sandler J. (2015). Emergencies in orthodontics. Part 1: Management of general orthodontic problems as well as common problems with fixed appliances. Dent. Update.

[6] Bradley E., Shelton A., Hodge T., Morris D., Bekker H., Fletcher S., Barber S. (2020). Patient-reported experience and outcomes from orthodontic treatment. J. Orthod..

[7] Romano F.L., Valério R.A., Gomes-Silva J.M., Ferreira J.T.L., Faria G., Borsatto M.C. (2012). Clinical Evaluation of the Failure Rate of Metallic Brackets Bonded with Orthodontic Composites. Braz. Dent. J..

[8] Ogiński T., Kawala B., Mikulewicz M., Antoszewska-Smith J. (2020). A Clinical Comparison of Failure Rates of Metallic and Ceramic Brackets: A Twelve-Month Study. BioMed Res. Int..

[9] Huilcapi M., Armas-Vega A., Cardenas A.F.M., Araujo L.C.R., Ocampo J.B., Bandeca M.C., de Siqueira F.S.F., Loguercio A. (2020). Effect of Surface Treatments on the Adhesive Properties of Metallic Brackets on Fluorotic Enamel. Dental Press J. Orthod..

[10] Almosa N., Zafar H. (2018). Incidence of orthodontic brackets detachment during orthodontic treatment: A systematic review. Pak. J. Med. Sci..

[11] Stasinopoulos D., Papageorgiou S.N., Kirsch F., Daratsianos N., Jäger A., Bourauel C. (2018). Failure Patterns of Different Bracket Systems and Their Influence on Treatment Duration: A Retrospective Cohort Study. Angle Orthod..

[12] Kafle D., Mishra R.K., Hasan M.R., Saito T. (2020). A Retrospective Clinical Audit of Bracket Failure among Patients Undergoing Orthodontic Therapy. Int. J. Dent..

[13] Alzainal A.H., Majud A.S., Al-Ani A.M., Mageet A.O. (2020). Orthodontic Bonding: Review of the Literature. Int. J. Dent..

[14] Bakhadher W., Halawany H., Talic N., Abraham N., Jacob V. (2015). Factors Affecting the Shear Bond Strength of Orthodontic Brackets—A Review of In Vitro Studies. Acta Med..

[15] Cossellu G., Lanteri V., Butera A., Sarcina M., Farronato G. (2015). Effects of Six Different Preventive Treatments on the Shear Bond Strength of Orthodontic Brackets: In Vitro Study. Acta Biomater. Odontol. Scand..

[16] Veli I., Akin M., Baka Z.M., Uysal T. (2016). Effects of Different Pre-Treatment Methods on the Shear Bond Strength of Orthodontic Brackets to Demineralized Enamel. Acta Odontol. Scand..

[17] Paschos E., Kurochkina N., Huth K.C., Hansson C.S., Rudzki-Janson I. (2009). Failure Rate of Brackets Bonded with Antimicrobial and Fluoride-Releasing, Self-Etching Primer and the Effect on Prevention of Enamel Demineralization. Am. J. Orthod. Dentofac. Orthop..

[18] Jurišić S., Jurišić G., Jurić H. (2015). Influence of Adhesives and Methods of Enamel Pretreatment on the Shear Bond Strength of Orthodontic Brackets. Acta Stomatol. Croat..

[19] Hamza B., Attin T., Cucuzza C., Gubler A., Wegehaupt F.J. (2020). RDA and REA Values of Commercially Available Toothpastes Utilising Diamond Powder and Traditional Abrasives. Oral Health Prev. Dent..

[20] Mason S., Young S., Araga M., Butler A., Lucas R., Milleman J.L., Milleman K.R. (2019). Stain control with two experimental dentin hypersensitivity toothpastes containing spherical silica: A randomised, early-phase development study. BDJ Open.

[21] Churchley D., Schemehorn B.R. (2013). In vitro assessment of a toothpaste range specifically designed for children. Int. Dent. J..

[22] Aydın B., Pamir T., Baltaci A., Orman M.N., Turk T. (2015). Effect of storage solutions on microhardness of crown enamel and dentin. Eur. J. Dent..

[23] Demirovic K., Slaj M., Spalj S., Slaj M., Kobaslija S. (2018). Comparison of Shear Bond Strength of Orthodontic Brackets Using Direct and Indirect Bonding Methods in Vitro and in Vivo. Acta Inform. Med..

[24] Scribante A., Gallo S., Turcato B., Trovati F., Gandini P., Sfondrini M.F. (2020). Fear of the Relapse: Effect of Composite Type on Adhesion Efficacy of Upper and Lower Orthodontic Fixed Retainers: In Vitro Investigation and Randomized Clinical Trial. Polymers.

[25] Wiegand A., Begic M., Attin T. (2006). In vitro evaluation of abrasion of eroded enamel by different manual, power and sonic toothbrushes. Caries Res..

[26] Scribante A., Dermenaki Farahani M.R., Marino G., Matera C., Rodriguez y Baena R., Lanteri V., Butera A. (2020). Biomimetic Effect of Nano-Hydroxyapatite in Demineralized Enamel before Orthodontic Bonding of Brackets and Attachments: Visual, Adhesion Strength, and Hardness in In Vitro Tests. BioMed Res. Int..

[27] Sfondrini M.F., Gallo S., Turcato B., Montasser M.A., Albelasy N.F., Vallittu P.K., Gandini P., Scribante A. (2021). Universal Adhesive for Fixed Retainer Bonding: In Vitro Evaluation and Randomized Clinical Trial. Materials.

[28] Mohammed R.E., Abass S., Abubakr N.H., Mohammed Z.M. (2016). Comparing orthodontic bond failures of light-cured composite resin with chemical-cured composite resin: A 12-month clinical trial. Am. J. Orthod. Dentofac. Orthop..

[29] Beltrami R., Chiesa M., Scribante A., Allegretti J., Poggio C. (2016). Comparison of shear bond strength of universal adhesives on etched and nonetched enamel. J. Appl. Biomater. Funct. Mater..

[30] Scribante A., Contreras-Bulnes R., Montasser M.A., Vallittu P.K. (2016). Orthodontics: Bracket Materials, Adhesives Systems, and Their Bond Strength. BioMed Res. Int..

[31] Poggio C., Scribante A., Della Zoppa F., Colombo M., Beltrami R., Chiesa M. (2014). Shear bond strength of one-step self-etch adhesives to enamel: Effect of acid pretreatment. Dent. Traumatol..

[32] Hajrassie M.K., Khier S.E. (2007). In-vivo and in-vitro comparison of bond strengths of orthodontic brackets bonded to enamel and debonded at various times. Am. J. Orthod. Dentofac. Orthop..

[33] Øgaard B., Fjeld M. (2010). The enamel surface and bonding in orthodontics. Semin. Orthod..

[34] Ladhe K.A., Sastri M.R., Madaan J.B., Vakil K.K. (2014). Effect of Remineralizing Agents on Bond Strength of Orthodontic Brackets: An in Vitro Study. Prog. Orthod..

[35] Al-Twaijri S., Viana G., Bedran-Russo A.K. (2011). Effect of prophylactic pastes containing active ingredients on the enamel-bracket bond strength of etch-and-rinse and self-etching systems. Angle Orthod..

[36] Ekizer A., Zorba Y.O., Uysal T., Ayrikcila S. (2012). Effects of Demineralizaton-Inhibition Procedures on the Bond Strength of Brackets Bonded to Demineralized Enamel Surface. Korean J. Orthod..

[37] Zarif Najafi H., Bagheri R., Pakshir H.R., Tavakkoli M.A., Torkan S. (2019). Effect of Different Surface Treatment on the Shear Bond Strength of Metal Brackets to Bleached and Desensitized Enamel. Int. Orthod..

[38] Cehreli S.B., Guzey A., Arhun N., Cetinsahin A., Unver B. (2010). The Effects of Prophylactic Ozone Pretreatment of Enamel on Shear Bond Strength of Orthodontic Brackets Bonded with Total or Self-Etch Adhesive Systems. Eur. J. Dent..

[39] Mahajan M., Singla A., Saini S.S. (2015). Comparative Evaluation of Different Prophylaxis Pastes on Shear Bond Strength of Orthodontic Brackets Bonded with Self Etch Primer: An in-Vitro Study. J. Indian Orthod. Soc..

[40] Rivera-Prado H., Moyaho-Bernal Á., Andrade-Torres A., Franco-Romero G., Montiel-Jarquín Á., Mendoza-Pinto C., García-Cano E., Hernández-Ruíz A.K. (2015). Efficiency in bracket bonding with the use of pretreatment methods to tooth enamel before acid etching: Sodium hypochlorite vs. hydrogen peroxide techniques. Acta Odontol. Latinoam..

[41] Soares F.M.Z., Follak A., da Rosa L.S., Montagner A.S., Lenzi T.L., Rocha R.O. (2016). Bovine tooth is a substitute for human tooth on bond strength studies: A systematic review and meta-analysis of in vitro studies. Dent. Mater..

[42] Sonesson M., Bergstrand F., Gizani S., Twetman S. (2017). Management of Post-Orthodontic White Spot Lesions: An Updated Systematic Review. Eur. J. Orthod..

[43] Al-Kawari H.M., Al-Jobair A.M. (2014). Effect of different preventive agents on bracket shear bond strength: In vitro study. BMC Oral Health.

[44] Montasser M.A., Drummond J.L. (2009). Reliability of the adhesive remnant index score system with different magnifications. Angle Orthod..

[45] Hitz Lindenmüller I., Lambrecht J.T. (2011). Oral care. Curr. Probl. Dermatol..

[46] Dominguez G.C., Tortamano A., Lopes L.V.D.M., Catharino P.C.C., Morea C. (2013). A Comparative Clinical Study of the Failure Rate of Orthodontic Brackets Bonded with Two Adhesive Systems: Conventional and Self-Etching Primer (SEP). Dental Press J. Orthod..

[47] Krishnan S., Pandian S., Rajagopal R. (2017). Six-Month Bracket Failure Rate with a Flowable Composite: A Split-Mouth Randomized Controlled Trial. Dental Press J. Orthod..

[48] Sukhia R.H., Sukhia H.R., Azam S.I., Nuruddin R., Rizwan A., Jalal S. (2019). Predicting the Bracket Bond Failure Rate in Orthodontic Patients: A Retrospective Cohort Study. Int. Orthod..

[49] Ravichandran N.K., Tumkur Lakshmikantha H., Park H.S., Jeon M., Kim J. (2019). Analysis of Enamel Loss by Prophylaxis and Etching Treatment in Human Tooth Using Optical Coherence Tomography: An In Vitro Study. J. Healthc. Eng..

[50] Burgess A.M., Sherriff M., Ireland A.J. (2006). Self-etching primers: Is prophylactic pumicing necessary? A randomized clinical trial. Angle Orthod..

[51] Sinjari B., d’Addazio G., Bozzi M., Santilli M., Traini T., Murmura G., Caputi S. (2019). SEM Analysis of Enamel Abrasion after Air Polishing Treatment with Erythritol, Glycine and Sodium Bicarbonate. Coatings.

[52] González-Cabezas C., Hara A.T., Hefferren J., Lippert F. (2013). Abrasivity testing of dentifrices—Challenges and current state of the art. Monogr. Oral.

